# Correlation study on gut microbiota and myosteatosis in patients with liver cirrhosis

**DOI:** 10.3389/fnut.2025.1513973

**Published:** 2025-02-04

**Authors:** Ninghui Zhao, Jinjia Bai, Xinmiao Li, Guofen Xu, Xiujuan Fu, Jing Li, Lingyun Niu, Jia Yao, Xiaoshuang Zhou

**Affiliations:** ^1^Department of Gastroenterology, Third Hospital of Shanxi Medical University, Shanxi Bethune Hospital, Shanxi Academy of Medical Sciences, Tongji Shanxi Hospital, Taiyuan, China; ^2^Department of Nephrology, The Affiliated People’s Hospital of Shanxi Medical University, Taiyuan, China

**Keywords:** liver cirrhosis, myosteatosis, gut microbiota, metagenomic sequencing, diagnostic value

## Abstract

**Objective:**

To investigate the features of gut microbiota in cirrhotic patients with myosteatosis and identify specific bacterial species that may be involved in the pathogenesis of myosteatosis.

**Methods:**

80 patients with liver cirrhosis were categorized into the myosteatosis group (*n* = 44) and the non-myosteatosis group (*n* = 36). Metagenomic sequencing was used to analyze the differences in gut microbiota composition between the two groups. Subsequently, the value of meaningful gut microbiota in the diagnosis of myosteatosis in patients with liver cirrhosis was analyzed.

**Results:**

At the species level, however, 15 bacterial species exhibited significant differences in relative abundance between these two groups. The relative abundance of *Roseburia hominis* and *Subdoligranulum unclassified* was inversely associated with mean muscle attenuation density at the L_3_ level (*p* < 0.05). Assessement of the diagnostic potential of *Roseburia hominis* and *Subdoligranulum unclassified* for the development of myosteatosis showed that the areas under the ROC curves (AUCs) was 0.869 [95% confidence interval (CI): 0.709–1.029; *p* < 0.05] for *Roseburia hominis* and 0.828 (95% CI: 0.6472–1.009; *p* < 0.05) for *Subdoligranulum unclassified*.

**Conclusion:**

Our study establishes compositional alterations of gut microbiota in patients with liver cirrhosis combined with myosteatosis and suggests the diagnostic potential for using gut microbiota as noninvasive biomarkers.

## 1 Introduction

Myosteatosis refers to the pathological deposition of fat in skeletal muscle due to metabolic dysregulation and compromised health status. It is often associated with a reduction in muscle strength, alterations in muscle architecture, impaired muscle contraction, and a decrease in muscle volume (1). Myosteatosis is a prevalent condition in cirrhotic patients and is correlated with an elevated risk of mortality, hepatic encephalopathy and portal hypertension in this patient population (2–4). Therefore, a better understanding of the factors for myosteatosis in cirrhotic patients will aid in multidisciplinary treatment of cirrhosis and improve patient prognosis.

However, the etiological factors contributing to myosteatosis remain unclear. Aging, metabolic disorders, non-metabolic conditions, and muscle injury may contribute to the initiation and progression of fat deposition in skeletal muscle, culminating in the development of myosteatosis (5–7). Recent evidence suggests a significant association between gut microbiota and lipid metabolism within skeletal muscle (8, 9). Tachi *et al.* demonstrated that myosteatosis was a significant factor for the development of sarcopenia in cirrhotic patients (10). Furthermore, di Cola *et al*. found that myosteatosis was more prevalent among patients with cirrhosis and was associated with an elevated mortality rate (11). Among cirrhotic patients, alterations in the composition of gut microbiota and small intestinal bacterial overgrowth (SIBO) play pivotal roles in the pathogenesis of sarcopenia (3, 12, 13). Moreover, changes in body composition were significantly correlated with modifications in the gut microbiome in individuals with cirrhosis (14). However, no study has explored the interactions between gut microbiota and myosteatosis in patients with liver cirrhosis. Hence, in the present study we measured the mean muscle attenuation density at the third lumbar vertebra (L_3_) level to assess myosteatosis in patients with liver cirrhosis, and used metagomic sequencing techniques to obtain gut microbiota information, in an attempt to investigate the potential correlation between myosteatosis and gut microbiota in this patient population.

Currently, the gut microbiome is emerging as a promising predictive biomarker for a range of diseases. Evidence indicates that the gut microbiome is highly precise used in the detection and grading of liver diseases (15). Thus, we further assessed the potential diagnostic utility of specific bacterial species as biomarkers for myosteatosis in cirrhotic patients, so as to offer a theoretical basis for the application of targeted microorganisms and their derivatives to improve prognosis, decrease mortality, and facilitate multidisciplinary management of cirrhosis.

## 2 Materials and methods

### 2.1 Patients

The clinical data of 80 patients with liver cirrhosis admitted to Shanxi Bethune Hospital between January 2023 and June 2023 were retrospectively analyzed. There were 60 males and 20 females, with an average age of (55.9 ± 11.2) years. This study was approved by the Shanxi Medical University Medical Ethics Committee. All work was carried out in compliance with the Ethical Principles for Medical Research Involving Human Subjects outlined in the Helsinki Declaration in 1975 (revised in 2000). All research was conducted in accordance with both the Declarations of Helsinki and Istanbul. All participants signed written informed consent forms.

The inclusion criteria were as follows: (i) with liver cirrhosis confirmed by imaging studies, biochemical markers, clinical assessment, and pathological findings; (ii) no antibiotics or probiotics had been used within 4 weeks preceding stool sample collection, and there was no history of fecal microbiota transplantation; (iii) having undergone abdominal computed tomography (CT) scans; and (iv) there was no record of gastrointestinal hemorrhage within the 4 weeks prior to enrollment.

The exclusion criteria were: (i) with co-existing chronic diarrhea, inflammatory bowel disease, or other conditions that can cause gut dysbiosis; (ii) with co-morbid severe dysfunction of other organs, excluding hepatic encephalopathy, hepatopulmonary syndrome, or hepatorenal syndrome; (iii) with a history of gastrointestinal surgery; (iv) with primary liver tumors/extrahepatic malignancies or having undergone liver transplantation; (v) with other malignancies; and (vi) pregnant or lactating women.

### 2.2 Assessment of myosteatosis

As muscle density attenuation value can serve as a crucial criterion for myosteatosis diagnosis (16), we determined the mean Hounsfield Unit (HU) value of muscle density attenuation across the entire muscle region utilizing transverse computed tomography (CT) scan images at the L_3_ level and analyzed the results with the SliceOmatic 5.0 software. Myosteatosis was defined based on mortality-associated thresholds established in a prior study: the muscle density attenuation value is <41 HU for individuals with a body mass index (BMI) below 24.9 kg/m^2^ and less than 33 HU for those with a BMI of ≥25 kg/m^2^ (17).

Since patients with liver cirrhosis are often accompanied by ascites and edema, which affects the BMI value, the dry body mass BMI (dry body weight / height^2, kg/m^2^) was used in this study. Dry weight (DW) can be evaluated or calculated using the following methods: (i) body mass before fluid retention; (ii) body mass after puncture and drainage; (iii) correction of body mass: Correction was made by subtracting a certain amount of body mass according to the severity of ascites judged clinically (mild 5%, moderate 10%, severe 15%, and subtract 5% if peripheral edema is present).

### 2.3 Fecal DNA extraction

Fresh stool samples were collected in the morning, deposited into sterile stool collection containers, and subsequently transferred to a-80°C freezer within 3 h for cryopreservation. Fecal DNA extraction was performed in accordance with the manufacturer’s protocol utilizing the QIAamp PowerFecal Pro DNA Kit. The purity and integrity of the extracted DNA were assessed by agarose gel electrophoresis (AGE), and the DNA concentration was accurately quantified using the Qubit system.

### 2.4 Metagenomic sequencing

Metagenomic sequencing was conducted according to the kit instructions [KAPA Hyper-Plus PCR-Free Kit (96-rxn)]. DNA samples that met the quality criteria were subjected to shearing into a target size of approximately 350 base pairs using a Covaris ultrasonic disruptor. Subsequently, the entire library was constructed through a series of steps including end-repair, A-tailing, adapter ligation, purification, and PCR amplification. Following the completion of library construction, initial quantification was performed using Qubit 2.0. The library was then diluted to a concentration of 2 ng/μL, and the insert size was detected using the Pcarey qSep100 system. Upon confirmation of the expected insert size, the effective concentration of the library was precisely determined using quantitative PCR (Q-PCR) with a target library concentration greater than 3 nM, so as to ensure the library quality. Illumina HiSeq sequencing was conducted for the qualified library. DNA polymerase and 4 kinds of fluorescently labeled dNTP were added to the flow cell, incorporating only a single base per cycle; the laser scanned the surface of the flow cell, captured the fluorescence signal, and read the nucleotide species on each template sequence polymerization; the “fluorophores” and “terminating groups” were chemically cut to restore the 3′ end viscosity and continue to incorporate a second round of single base; the results of fluorescence signals collected in each round were sequentially counted to obtain the sequence of template DNA fragments.

All the raw metagenomic sequencing data underwent quality control (QC) utilizing the MOCAT2 software package (18). SOAPdenovo^1^ was employed for further analysis of the QC’d metagenomic sequencing data. Scaffold sequences (scaftigs) were derived from the assembly output, and gene structures were predicted using MetaGeneMark. The predicted genes were clustered and reduced redundancy using CD-HIT. Genes with greater than 95% identity over 90% of the shorter gene length were clustered together. The longest sequence within each cluster was selected as the representative sequence. A non-redundant gene set was constructed with genes longer than 100 base pairs. At the gene level, high-quality reads were aligned to this non-redundant reference gene catalog using BWA. Reads shorter than 30 base pairs and those below 95% identity were discarded. The read count for each gene was tallied and normalized, thus obtaining the relative abundance of the genes. At the species level and other higher taxonomic ranks, MetaPhlAn 2.0 was employed to determine the relative abundance of the microbiota.

### 2.5 Statistical analysis

Since the relative abundance of gut microbiota did not conform to normal distribution, nonparametric Kruskal-Wallis rank-sum test was utilized to identify differences in the gut microbiota between the myosteatosis and non-myosteatosis groups. Additionally, LEfSe was employed to compare the biome data between these two groups. SPSS version 26.0 was used for the statistical analysis of demographic data, clinical features, and laboratory findings of the enrolled patients. The count data are expressed as percentages and compared with Chi-square test. The measurement data are presented using ^−^X ± SD and compared using *t* test. A *p* value <0.05 was considered statistically significant. The value of gut microbiota that had been found to be significant for the diagnosis of myosteatosis in patients with liver cirrhosis was analyzed using receiver operating characteristic (ROC) curves.

## 3 Results

### 3.1 Patient characteristics

Patients with liver cirrhosis were stratified into two groups based on muscle density attenuation at the L_3_ level, resulting in 44 patients in the myosteatosis group and 36 patients in the non-myosteatosis group. No significant differences in age and gender distribution were observed between these two groups. The muscle density attenuation was (34.6 ± 5.6) HU in the myosteatosis group, which was significantly lower than that [(40.8 ± 4.2) HU] in the non-myosteatosis group (*p* < 0.05). No significant differences were observed in blood biochemical markers, ascites, and Child-Pugh scores (Table 1).

**Table 1:** Comparison of clinical data between myosteatosis group and non-myosteatosis group (^−^X ± SD, %).

	Myosteatosis group (*n* = 44)	Non-myosteatosis group (*n* = 36)	*p*- value
Age (years)	58.36 ± 9.19	53.00 ± 13.18	0.298
Gender (female)	8 (22.2)	12 (27.3)	0.604
Etiology (*n*, %)			0.994
HBV	24 (54.5)	19 (52.8)	
HCV	11 (25.0)	10 (27.8)	
Alcohol	5 (11.4)	4 (11.1)	
Others	4 (9.1)	3 (8.3)	
Muscle density attenuation (HU)	34.61 ± 5.61	40.80 ± 4.23	0.014
BMI	22.85 ± 3.39	25.54 ± 4.68	0.163
ALT (U/L)	43.17 ± 32.49	56.90 ± 63.99	0.541
AST (U/L)	54.36 ± 36.70	68.93 ± 80.68	0.598
TBIL (μmol/L)	44.01 ± 52.67	39.02 ± 37.85	0.815
INR	1.33 ± 0.25	1.61 ± 0.61	0.216
Albumin (g/L)	33.02 ± 8.49	31.10 ± 4.30	0.523
Urea (mmol/L)	4.45 ± 1.67	5.51 ± 1.76	0.226
Creatinine (μmol/L)	65.06 ± 11.57	73.57 ± 11.61	0.128
Ascites (*n*, %)	24 (54.5)	20 (55.6)	0.928
Child-Pugh score	10.09 ± 1.64	10.44 ± 1.81	0.653

HBV, hepatitis B virus; HCV, hepatitis C virus; BMI, body mass index; ALT, alanine aminotransferase; AST, aspartate transferase; TBIL, total bilirubin; INR, international normalized ratio.

### 3.2 Results of differential abundance analysis

**Figure 1:**
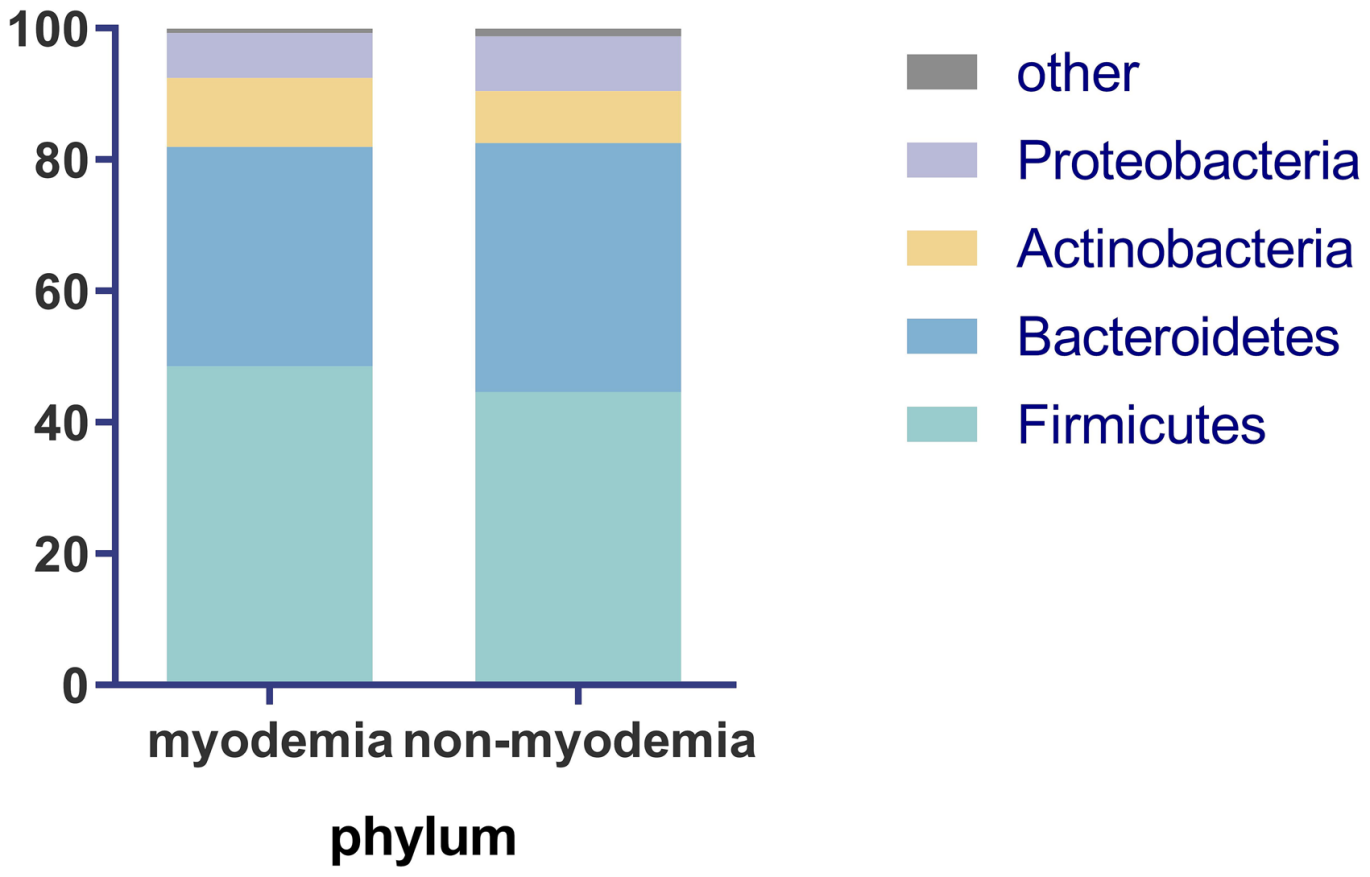
Comparison of microbiota abundance at phylum level between the myosteatosis and non-myosteatosis groups.

At the phylum level, the predominant bacterial taxa in the fecal microbiota of cirrhotic patients, irrespective of myosteatosis status, were *Firmicutes* and *Bacteroidetes*, followed by *Actinobacteria* and *Proteobacteria*. The myosteatosis and non-myosteatosis groups showed no significant differences in the relative abundance of gut microbiota, including *Firmicutes* (48.6% ± 13.5% versus 44.6% ± 20.2%), *Actinobacteria* (10.5% ± 16.3% versus 7.9% ± 11.4%), *Bacteroidetes* (33.4% ± 21.1% versus 37.9% ± 23.6%), and *Proteobacteria* (6.8% ± 9.4% versus 8.3% ± 8.4%; all *p* > 0.05; Figure 1).

**Figure 2:**
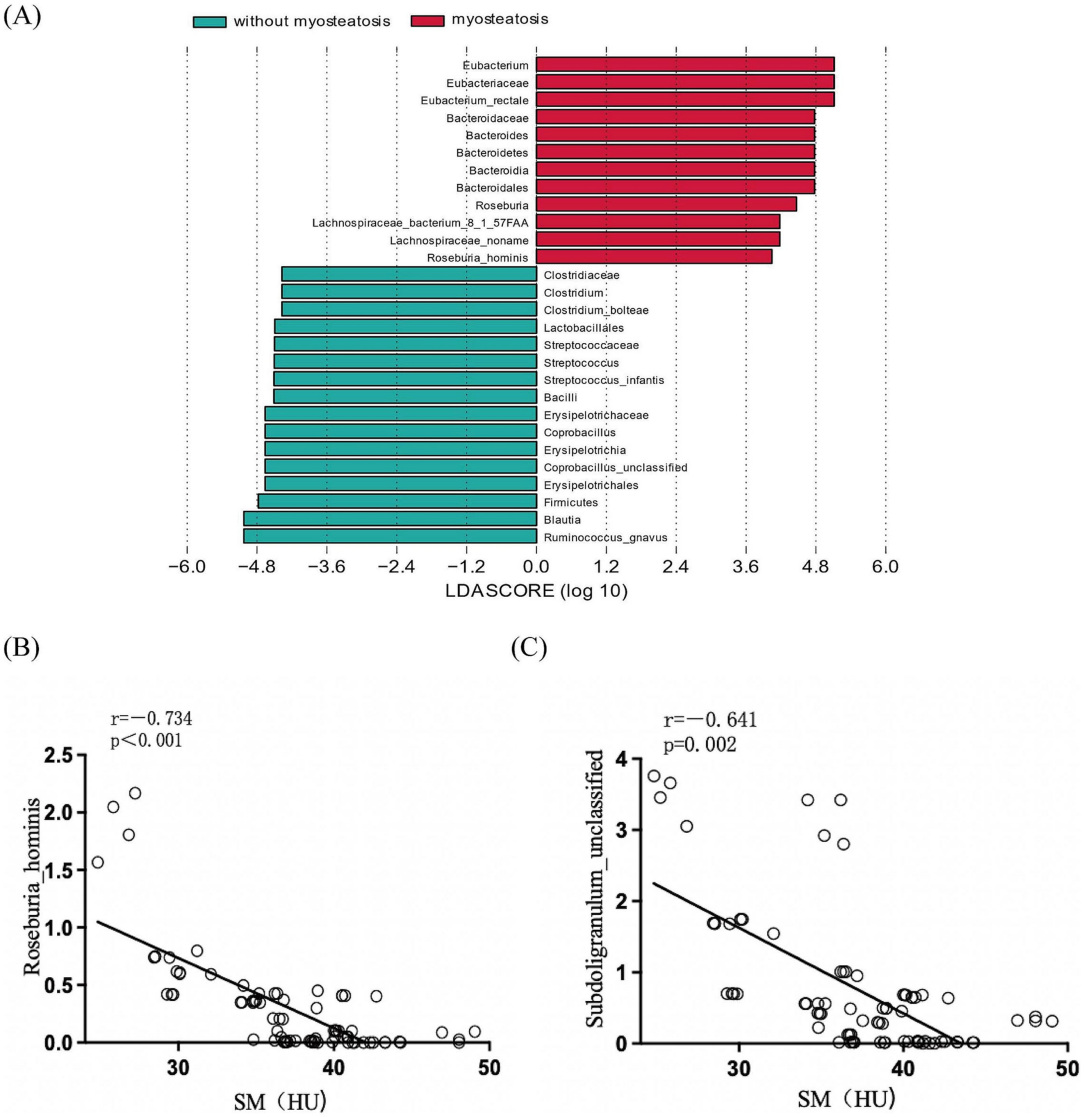
**(A)** Comparison of the relative abundance of gut microbiota at the species level between the myoseatosis and non-myosteatosis groups. Green: non-myosteatosis group; red: myosteatosis group. **(B)** Correlation analysis of *Roseburia hominis* and muscle density attenuation at the L_3_ level. **(C)** Correlation analysis of Subdoligranulum unclassified and muscle density attenuation at the L_3_ level.

The nonparametric Kruskal-Wallis rank-sum test comparing gut microbiota composition at the species level revealed significant differences in the relative abundance of 15 bacterial species between the myosteatosis and non-myosteatosis groups (all *p* < 0.05). Specifically, the relative abundance of *Coprobacillus unclassified*, *Clostridium bolteae*, and *Streptococcus infantis* was significantly reduced in the myosteatosis group, whereas the relative abundance of *Faecalibacterium prausnitzii, Eubacterium rectale, Bacteroides stercoris, Ruminococcus gnavus, Roseburia inulinivorans, Bacteroides uniformis, Subdoligranulum unclassified, Ruminococcus lactaris, Dorea formicigenerans, Bacteroides cellulosilyticus,* and *Roseburia hominis* were significantly increased (all *p* < 0.05; Figure 2A). Furthermore, correlation analyses between these significantly different microbial species and the muscle density attenuation at the L_3_ level showed that *Roseburia hominis* (*r* = −0.734, *p* < 0.001) *and Subdoligranulum unclassified* (*r* = −0.641, *p* = 0.002) were negatively correlated with the muscle density attenuation at the L_3_ level (Figures 2B,C).

### 3.3 Potential diagnostic value of relevant bacterial strains for myosteatosis in cirrhotic patients

**Figure 3:**
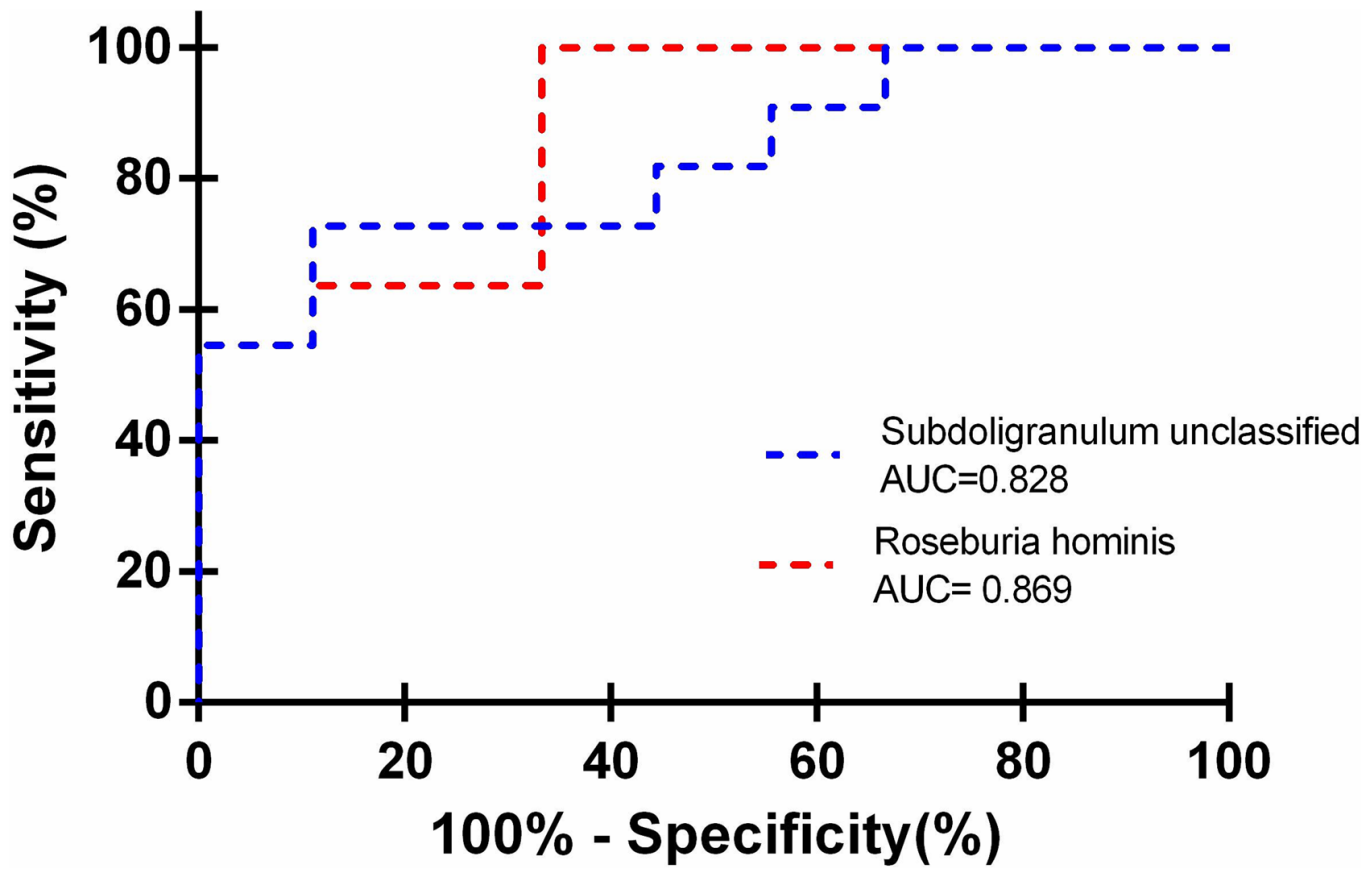
Utilization of *Roseburia hominis* and *Subdoligranulum unclassified* as predictors for myosteatosis in cirrhotic patients.

Subsequently, we assessed the potential diagnostic utility of *Roseburia hominis* and *Subdoligranulum unclassified* in cirrhotic patients using receiver operating characteristic (ROC) curves, which yielded an area under the curve (AUC) of 0.869 (95% CI: 0.709–1.029; *p* = 0.006), a Youden index of 0.587, a sensitivity of 92%, and a specificity of 66.7% for *Roseburia hominis* and an AUC of 0.828 (95% CI: 0.6472–1.009; *p* = 0.013), a Youden index of 0.616, a sensitivity of 72.7%, and a specificity of 88.9% for *Subdoligranulum unclassified*. These findings suggested that both *Roseburia hominis* and *Subdoligranulum unclassified* had diagnostic potential for the development of myosteatosis in cirrhotic patients (Figure 3).

## 4 Discussion

Gut microbiota is intricately associated with myosteatosis. In the present study, metagenomic sequencing was employed to analyze the characteristics of gut microbiota in patients with liver cirrhosis. No substantial differences in gut microbial composition at the phylum level were found between myosteatosis and non-myosteatosis groups. At the species level, however, 15 bacterial species exhibited significant differences in relative abundance between the myosteatosis and non-myosteatosis groups. Among them, *Roseburia hominis* and *Subdoligranulum unclassified* were identified as being negatively correlated with muscle density attenuation at the L_3_ level, indicating their potential diagnostic utility for myosteatosis.

The microbial composition of patients with liver cirrhosis in our study was consistent with findings from previous research, with *Firmicutes, Bacteroidetes, Actinobacteria,* and *Proteobacteria* as the dominant bacterial phyla (19). No significant differences were found in gut microbial composition at the phylum level between myosteatosis and non-myosteatosis groups. On the one hand, the individual gut microbiota is stable and conservative at the phylum level. On the other hand, studies have shown that gut microbiota diversity correlates with the severity of cirrhosis (20). Prior research indicated that there was an increase in the abundance of *Bacteroides* in patients with compensated liver cirrhosis, which decreased to normal levels upon the onset of decompensated cirrhosis (21). The present study enrolled both compensated and decompensated cirrhosis patients. These two subpopulations had similar clinical conditions, which may have resulted in the lack of a significant difference in the gut microbiota at the phylum level. In order to further explore the effect of intestinal microbiota on myosteatosis in liver cirrhosis, we further analyzed at the species level.

At the species level, we identified 15 bacterial species that displayed significant differences in relative abundance between the myosteatosis and non-myosteatosis groups. Among them, *Roseburia hominis* and *Subdoligranulum unclassified* were inversely correlated with muscle density attenuation at the L_3_ level. *Roseburia hominis* is a member of the genus *Roseburia*, characterized by its slightly curved rod shape and its involvement in carbohydrate metabolism within human gut microbiota through multiple flagellar movement. It was found that the abundance of *Roseburia hominis* was increased in patients with type 2 diabetes (22), and the incidence of myosteatosis was notably higher in individuals with type 2 diabetes compared to healthy individuals (23). In Singapore, the abundance of *Roseburia hominis* was found to be higher in older adults than in younger adults (24). Furthermore, myosteatosis was frequently detected in the elderly and was associated with myasthenia (25). *R. hominis* is characterized by the production of short-chain fatty acids (SCFA), SCFAs have been shown to influence lipid, carbohydrate and protein metabolism in skeletal muscle tissues both *in vitro* and *in vivo* (26). However, the mechanism of action of *Roseburia hominis* in myosteatosis in patients with cirrhosis still needs to be further explored. As myosteatosis often co-existed with a higher abundance of *Roseburia hominis*, we assumed that myosteatosis in patients with cirrhosis might be correlated with the increased abundance of *Roseburia hominis*.

Furthermore, we assessed the diagnostic potential of *Subdoligranulum unclassified* and *Roseburia hominis* for the detection of myosteatosis in patients with liver cirrhosis. The AUC was 0.828 (95% CI: 0.6472–1.009) for *Subdoligranulum unclassified* and 0.869 (95% CI: 0.709–1.029) for *Roseburia hominis*, showing high diagnostic value for myosteatosis. In fact, gut microbiome has demonstrated potential as a predictive biomarker for a spectrum of diseases (15). A composite of four bacterial genera — Veillonella, Lactobacillus, Spirillum, and Clostridium — discriminated patients with autoimmune hepatitis (AIH) from healthy individuals, highlighting the potential diagnostic significance of specific gut microbiota profiles in patients with AIH (27). Loomba R *et al*. have demonstrated that microbial markers can be utilized for the diagnosis of metabolic and fibrotic liver diseases, serving as an adjunct to invasive methods in determining the current stage of a liver disease (28). Caussy et al. used a random forest classifier model to discriminate non-alcoholic fatty liver disease (NAFLD) cirrhosis from non-NAFLD healthy controls based on 16S sequencing for gut-microbiome compositions (29). They found that the biomarkers derived from the random forest classifier model exhibited excellent diagnostic accuracy in predicting liver fibrosis. At present, the diagnosis of myosteatosis is mainly based on the calculation of muscle density attenuation by CT examination, which is affected by ascites or edema, and the CT examination is radiative. In the present study, we provided a fecal-based noninvasive test by using metagenomic sequencing and discovered that the bacterial species *Subdoligranulum unclassified* and *Roseburia hominis*, which were significantly linked to muscle density attenuation at the L_3_ level, possessed a high diagnostic value for the development of myosteatosis in patients with liver cirrhosis, confirming that gut microbiota may serve as potential biomarkers for the identification of complications in cirrhotic patients. In the future, it is anticipated that a predictive model based on gut microbiota composition will be developed to forecast the progression of liver disease and the onset of complications. It also provides a basis for the use of targeted microorganisms and their preparations to improve the poor prognosis of patients with liver cirrhosis. However, it is undeniable that the application of microbiota metagenomic sequencing-based diagnostics in the clinical setting has certain limitations. Due to the complexity of the analysis, trained personnel in sample handling are required to avoid errors and cross-contamination. In addition, instrumentation and reagents are costly, although there has been a substantial cost reduction in sequence data generation.

In addition, there is growing evidence that intestinal flora disturbances can affect alterations in the physiology and function of skeletal muscle. In patients with cirrhosis, it is often accompanied by disturbances of the intestinal flora and its metabolites (30). The pathology of the gut microbiota influences the development of sarcopenia (gut-muscle axis) in patients with cirrhosis. Decreased barrier function of the gut and liver, gut dysbiosis, and small intestinal bacterial overgrowth (SIBO) can lead to increased blood levels of ammonia, lipopolysaccharides, pro-inflammatory mediators, and myostatin. These factors have complex negative effects on muscle mass and function (31). Studies have demonstrated that myosteatosis is associated with increased systemic inflammation and insulin resistance. On the one hand, the disordered gut microbiota induces the immune system or through the abnormal production of metabolites such as endotoxins, leading to systemic inflammatory responses. Systemic inflammation exacerbates muscle wasting and fat infiltration (32). On the other hand, studies have shown that gut microbiota disturbances and liver cirrhosis may contribute to insulin resistance (33, 34). Some studies indicate that prebiotic supplementation increases skeletal muscle mass in obese mice and improves hand-grip strength in elderly humans (35, 36). Interventions targeting the gut microbiota had a positive impact on most segments of the damaged intestinal muscle axis in patients with cirrhosis. Therefore, management of patients with cirrhosis based on microbiota intervention is of great significance to guide their diagnosis and treatment.

Our present study has several limitations. Firstly, there is a paucity of research on the mechanisms by which gut microbes influence muscle mass. Further investigations are required to elucidate the specific ways in which microbial species contribute to energy metabolism, liver inflammation, and regulation of serum metabolites. Secondly, being a single-center study, due to its small sample size, we only investigated the correlations (rather than causal relationships) between gut microbiota and myosteatosis. Studies with larger sample sizes are warranted to explore whether interventions targeting gut microbiota can lower the incidence of myosteatosis in patients with liver cirrhosis to explore its therapeutic value, and to further reveal the causal relationships by integrating multi-omics and other methods. Thirdly, methods for assessing myosteatosis have limitations. While CT imaging can measure muscle density at L3, it does not accurately quantify fatty infiltration. In populations with cirrhosis, reduced muscle density could be due to either excess intramuscular fat or the presence of edema. Lastly, the present study did not investigate myosteatosis and the poor prognosis of liver cirrhosiss. Some studies showed that myosteatosis is associated with poor prognosis of cirrhosis, possibly because myosteatosis increases the complications of cirrhosis (3, 37). In addition, the absence of data on muscle function tests or frailty assessments is particularly noteworthy, as these could help clarify the critical role of muscle composition. Therefore, the exact molecular signaling pathways linking myosteatosis and poor prognosis for cirrhosis is unclear and needs to be further explored.

In conclusion, our study establishes compositional alterations of gut microbiota in patients with liver cirrhosis combined with myosteatosis and suggests the potential of the gut microbiota as a noninvasive biomarker for the diagnosis and treatment of myosteatosisin patients with liver cirrhosis.

## Data Availability

The original contributions presented in the study are included in the supplementary material, further inquiries can be directed to the corresponding authors.

## Footnotes

^1^version 2.04, parameters: all-D1 -M3-L 500, available at http://soap.genomics.org.cn
